# Analysis of Properties and Macroscopic Defects of Metallic Bars, Pipes, and Strands through the Spectrum of Low-Frequency Excitations

**DOI:** 10.3390/ma17102171

**Published:** 2024-05-07

**Authors:** Matteo Mancini, Bruno Turchetta, Matteo Cirillo

**Affiliations:** 1 Multiservizi Integrati srl, Via Caposile, 00195 Roma, Italy; mancini@topphysio.it (M.M.); brunoturchetta@topphysio.it (B.T.); 2 Dipartimento di Fisica and MINAS Lab, Università di Roma “Tor Vergata”, 00133 Roma, Italy

**Keywords:** acoustic resonance, piezoelectric sensors, materials physics

## Abstract

It is demonstrated that the application of piezoelectric sensors to metallic bars and strands can enable determining the status of the integrity of these elements through the spectrum of their acoustic excitations. The voltage output of the piezo, secured to metal bars or strands, is fed to the input of a Fast Fourier Transform analyzer, which allows displaying the spectrum of the excitations from which information on the length, overall quality of the metal, and the presence of defects can be obtained. We show that the analysis, performed on several materials and strands of different lengths, could be useful for cases in which visible inspection and/or direct access to the entire body of the metallic elements is not possible. Applications of our study for testing metallic structures embedded in concrete foundations are reported for construction sites.

## 1. Introduction

The phenomenon of resonance has opened relevant perspectives both at fundamental and applied levels in many fields of classical and quantum physics. A good starting point for obtaining insight into the topic is the 23rd chapter of the first volume of the Feynman Lectures on Physics [1]. It would be too long to address all of the aspects motivating the interest in this fundamental topic here. We will be concerned in this work with possible applications of acoustic resonances in metallic and solid-state media, which, in the limit of one-dimensional propagation, reduce to the classical patterns of vibrating strings [2,3]. The spectrum of the acoustic (low frequency) excitations allows characterizing, with remarkable uncertainty, a relevant physical characteristic of the media, namely, the sound propagation velocity. When visual and/or unconstrainted access to the metallic elements under investigation is possible, the acoustic characterization represents a viable way to check the quality of the material; however, the spectrum of the acoustic excitations can be especially helpful, if not unique, when accessing the metallic elements is obstructed and no visual access and/or direct inspection of these is possible.

**Figure 1:**
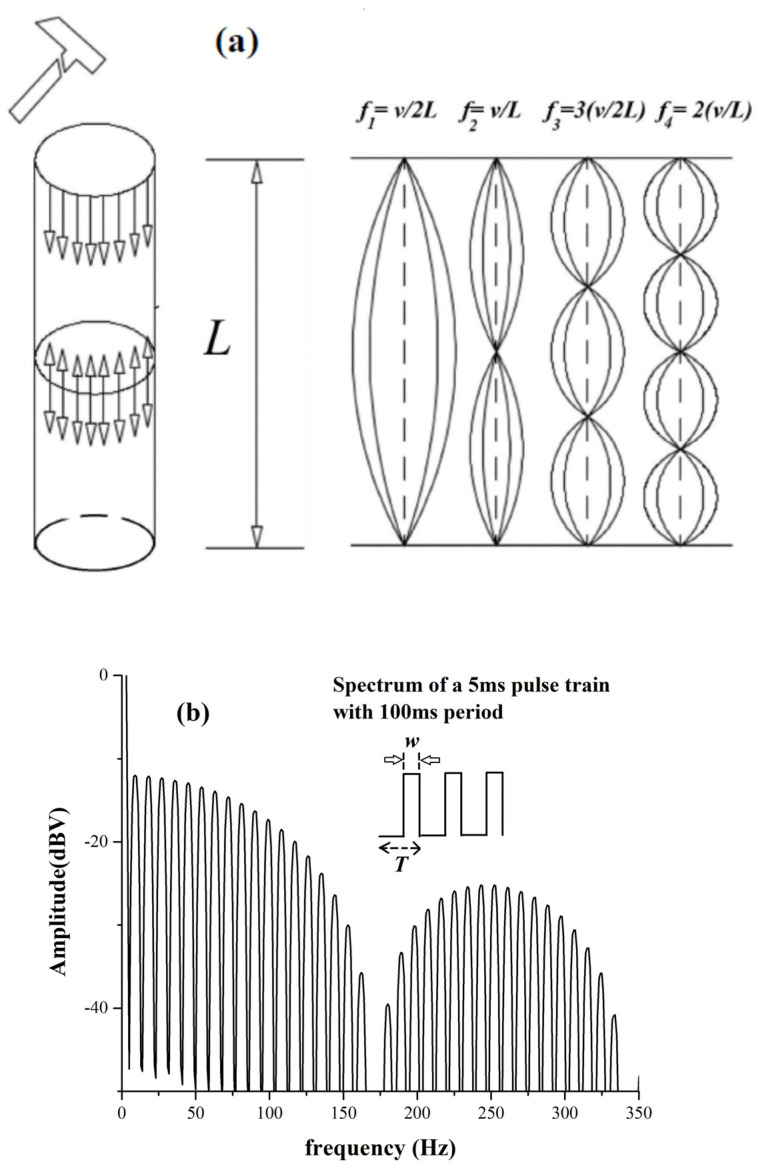
(**a**) Sketch of the acoustic excitations of a “regular” solid metallic bar: we show four harmonics in a vibrating string oscillating pattern and (**b**) the frequency spectrum of excitations generated by a train of pulses (inset), mimicking the signal detected in one point of the bar after the percussive excitation generating the pattern in (**a**). We set *w* (pulse width) = 5 ms and *T* (period) = 100 ms.

In Figure 1, we have sketched a bar of length *L*, and the hammer at one end mimics the possibility that the bar is percussion excited from one end to generate vibrations, which then travel along the bar itself. Considering the ends of the metal as free and considering only oscillations taking place along the “extended” dimension of the bar, the standing wave patterns sketched in the figure can take place. The “fundamental” frequency (or first harmonic) of the oscillations in the block will be *f* = *v*/*2 L*, where *v* is the propagation velocity of the acoustic vibrations in the bar. The figure shows the first harmonic excitation (also called fundamental mode), as well as the four successive ones characterized by frequencies *f* = *n v*/*2 L* (*n* = 1,2,3,4 in this specific case). When the bar under investigation only has one physical dimension that is “extended”, namely, much larger than the other two and of the same order of magnitude of acoustic wavelengths in the material, then the vibrating string standing wave pattern can be assumed as a reasonable model for the acoustic modes generated by material reticle transmission along the bar. In the next section, we shall see that this is often the case for several “one-dimensional” posts of metals. We note that equations for the frequencies that we have so far written only apply to posts and strands with material uniformly distributed along their “continuous” section. When the materials contain defects, breaks, or other types of irregularities, the above-written equations do not hold, and the acoustic spectrum may contain spurious harmonics or anomalous background noise levels. This is indeed one motivation for our work: when direct inspection of the status of the bar is not possible, the analysis of its acoustic spectrum can provide information on the status of its conservation. Nevertheless, we will not consider herein the case of metallic pipes that are empty inside since in this case, tracking all the acoustic modes can be more complex; however, we will also show in Section 4 the result of a measurement performed on a pipe filled with concrete.

It is worth recalling that there exists a “complementary” way to look at the analysis of the phenomenon shown in Figure 1a. If we think of a detector (a piezoelectric like in our case) sticking at one point of the bar, say at one end, it will be subject to pulses each time the wave is reflected from the end in a way that the resulting waveform in the time domain shall be a sequence of pulses spaced *T* = *2 L/v*. In the frequency domain, such waveforms will generate a spectrum containing harmonics spaced *v/2 L = 1/T*, as shown in Figure 1b, for a signal synthesizer-generated pulse train having a *T* = 100 ms period and a pulse width of 5 ms. We see that the spectrum is a sequence of 10 Hz (1/100 ms) spikes. The modulation of the amplitude of the harmonics is a fundamental consequence of Fourier analysis [4]. We will also see a typical example in our results.

This study of acoustic excitations has contributed to a wide extent to the understanding of the physical properties of solids [3], and it is the purpose of the present work to show that even macroscopic defects and properties of the solids can be investigated by the help of low-frequency oscillations and their related spectrum. Our approach somehow frames in the kind of testing and analysis of structures and materials that are non-destructive [5]. Within this framework, the low-frequency investigation of concrete blocks has gathered much attention [5,6,7], as has the use of piezoelectric sensors as transducers. Work has been presented concerning piezoelectric transducer calibration [8], and papers have concentrated on detecting defects in concrete steel bars by piezoelectric sensors [9,10,11]. The Electro-Mechanical Impedance (EMI) technique is very much considered for detecting defects in construction and related materials [12,13] and relies on low-frequency characterizations obtained by piezo sensors sticking on structures. Problems of post-tensioned tendons have also been investigated by piezo transducers [14], and a review reports on these developments, along with other diagnostic techniques [15].

In this paper, we present an alternative approach to investigate the quality of metallic bars and strands that merges together the possibilities offered by very sensitive piezoelectric sensors and the principle of wave/pulse propagation/reflections in one-dimensional materials. The main difference between our approach and previous investigations consists of the fact that we do not use any external AC source of excitation. We excite autonomous vibration modes and detect the spectrum with a very sensitive piezo sensor. From the “technical” point of view, this could be a complementary protocol to determine the size and location of defects, which could then be used for more detailed investigations. Nevertheless, the results that we present are also intriguing from the fundamental point of view inasmuch as the observed phenomenology is analogous to the phenomena observed coupling nonlinear transmission lines, in which pulses are transmitted and reflected through inductive or capacitive discontinuities [16,17,18]. Wave and pulse propagation in finite-size one-dimensional structures and reflections of waves at discontinuities are sources of interesting effects in classical and quantum physics and engineering. Our work also focuses on the general field of the application of resonances, a field that has known no expansion limits, so far. Recently, it has been shown that detailed studies of low-frequency vibrations can be very helpful in characterizing the performances of complex mechanical devices [19,20].

The remainder of the paper is structured as follows. In the next section, we report the measurements that we have performed on sample metallic bars made of different materials. We show how the acoustic spectrum of the excitations allows determining the propagation velocity for each specific material; in Section 3, we report the measurements we have used to characterize bars presenting defects or discontinuities and show how changes in the structural properties of the elements investigated in Section 3 can lead to a modification of the acoustic spectrum. In Section 4, we discuss and illustrate practical applications of our method to real posts employed in the construction engineering of buildings and highways. In Section 5, we draw the conclusions of this paper.

## 2. Measurements on Bars and Strands of Different Materials

**Figure 2:**
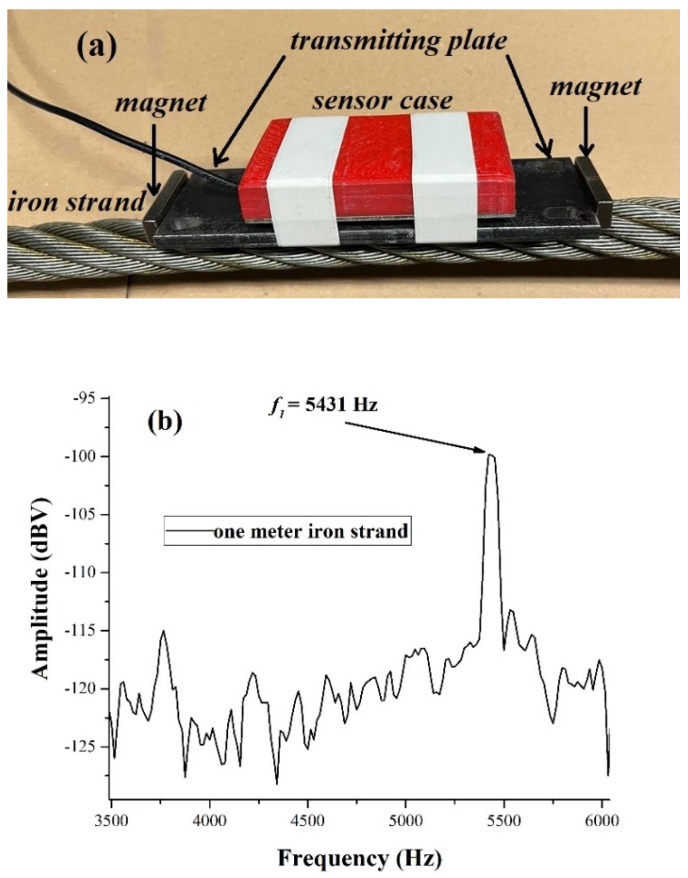
(**a**) The experimental setup that we use: the piezo sensor is inside the case and tied by robust tape to a transmission plate secured in turn to the iron strand by the magnets. Screws coming out of the sensor case “shorten” the piezo and transmitting plate. (**b**) The first harmonic (or fundamental) of an “open ended” one-meter strand. The strand diameter is 2 cm.

We first show and discuss sets of measurements of the sound propagation velocity in seven bars with cylindrical shapes made of different materials: anticorodal aluminum, brass, copper, iron, stainless steel, and iron strand. The bars are positioned horizontally, lying on acoustically isolating materials, and tiny percussive excitations, having an energy of the order of a few mJ, are generated at one end of the bar in order to generate the standing-wave patterns. The piezo sensor responsible for detecting the vibrations is secured to the bar far from the end where the light percussive excitation is generated, and the voltage output of it is sent to an FFT spectrum analyzer. Figure 2a shows how the piezo case is secured to a 2 cm in diameter iron strand. The case, a box sized 8 cm × 4 cm *×* 1 cm, is secured to the transmitting plate by robust tape and is shown in white in the figure. The piezo mount inside the case and its coupling couple it to the transmitting plate are now subject to a patent application.

The “transmitting” plate is secured to the bars by strong magnets; see Figure 2a for the iron strand. For non-magnetic materials, the plate is secured to the bar by strong adhesive tape or elastic rubber stripes. For magnetic metal bars, we checked that it made no difference in the piezo response when fixing the plate to the bars by magnets or by tape. Fixing with magnets was fast but also strong at the same time.

In Table 1, we report the value of the acoustic mode propagation velocity for the six different materials. In the last column, we show the values expected for the different materials [21,22]. As we see, our measurements provide a reasonable estimate of the propagation velocity. Several bars that we tested all had the same length (1.89 m) and all had the same section diameter (2 cm, except for copper). For the two iron strands, we see that the two measurements at 1.89 m and 1 m provide results consistent within 2%. We measured for the 1.89 m long strands a fundamental frequency of 2781 Hz, while for the one-meter strand, we measured, from the spectrum shown in a fundamental frequency, as shown in Figure 2b, values of 5431 Hz, which were consistent within the linewidths of the spectral lines (roughly 1% of the spectral lines).

## 3. Detecting Spectra Due to Wave Reflections and Resonances

**Figure 3:**
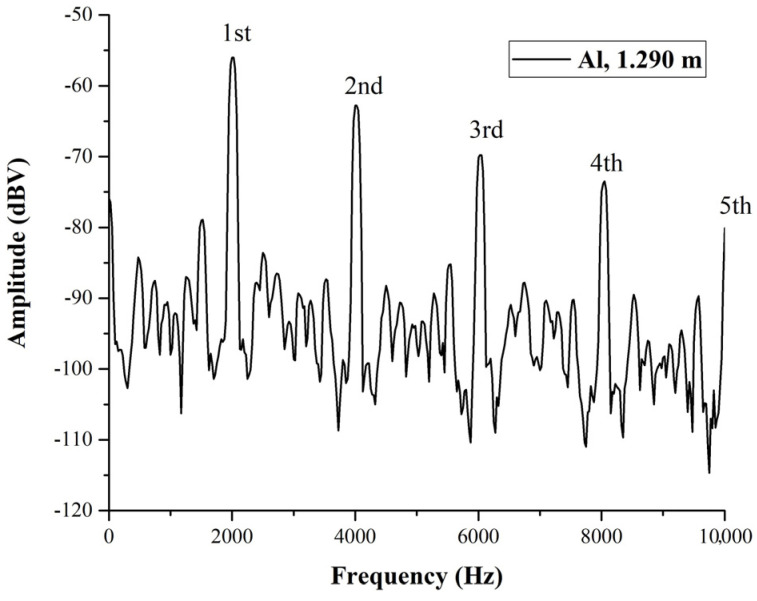
Acoustic spectrum of a 1.290 m long cylindrical anticorodal aluminum bar with a diameter of 0.035 m. We see the first harmonic at 2022 kHz and four more with a distance between them of 2.006 kHz. We also note that the sound velocity, in this case, is 5175 m/s, which is within 1.5% of the one reported in Table 1 for anticorodal aluminum; the discrepancy can be due to the fact that both the samples tested were “commercial” and not from the same batch. It is likely that the alloy composition was slightly different.

We are ready to discuss the possibility of detecting, by spectral analysis, defects in metallic posts and strands. In Figure 3, we show the acoustic spectrum of an anticorodal aluminum (an alloy of aluminum, silicon, magnesium, and manganese) bar that is 1.290 m long and has a diameter of 0.035 m. The spectrum is always obtained by generating a tiny percussive excitation with an energy of the order of a few mJ at one end of the bar. We can see clearly the fundamental frequency (or first harmonics) of the spectrum at 2022 Hz. From the length of the bar and the frequency of this fundamental excitation, inverting the equation for the frequency reported in the introduction, we obtain *v = 2 Lf* = 5175 m/s. In the spectrum, we can also see successive harmonics, which were, respectively, the second at 4028 Hz, the third at 6035 Hz, and the fourth 8041 Hz The fifth is expected at 10,047 (considering that the difference between the first four harmonics is 2006 Hz and, therefore, is within 0.5% from the last point of the horizontal (frequency) scale (10,000 Hz)).

**Figure 4:**
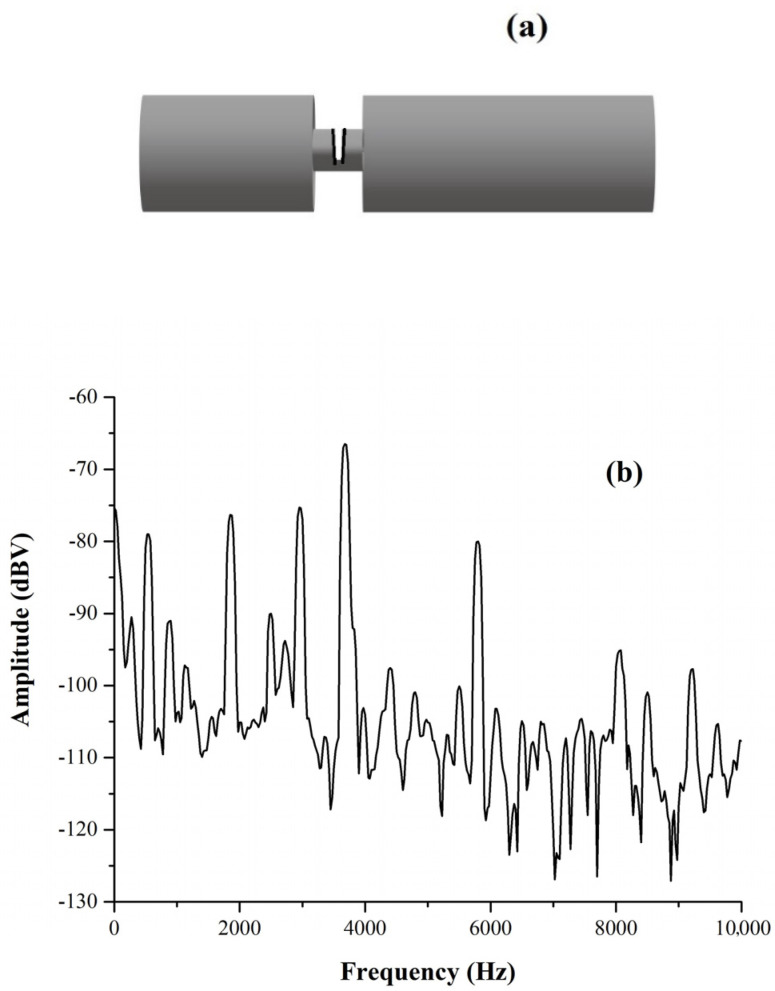
(**a**) (Not to scale.) The “defect” generated in a 1.29 m aluminum bar by a saw for metals after a regular groove had been generated by a lathe; (**b**) the spectrum generated by the defect in (**a**). The spectrum is substantially modified, and additional spectral components appear, and one in particular at 2950 Hz corresponds to having a resonance due to reflected waves at the defect (located at 0.880 m from one end of the bar).

The aluminum was then treated mechanically to generate a regular groove in it with a depth of 1 cm and a width of 0.5 cm at a distance of 0.88 m from one bar end (see Figure 4a), but the acoustic spectrum we obtained was not much different from that in Figure 3. In order to generate a more effective defect, we used a saw for metals producing an irregular crack in the aluminum bridge that we had previously obtained, as sketched in Figure 4a. The spectrum resulting from such a treatment was substantially modified with respect to the one shown in Figure 3, as we see in Figure 4b. Now, all over the frequency range (0–10 kHz), extra spectral components are visible, in addition to the second, third, and fourth harmonics of the bar.

In particular, a component raising more than 20 dBV above the noise level now exists at 2950 Hz. From this frequency, inverting the equation *f = v/2 L* for the length *L = v/2 f*, using the value *v* = 5175 m/s obtained before as the sound propagation velocity, we realized that the extra spectral component corresponded to a length *L* = 0.88 m, which was the distance from one end up to the point where the artificial defect was localized. The reflections of the standing waves due to the defect generate extra spectral components which, in turn, can allow identifying its position.

**Figure 5:**
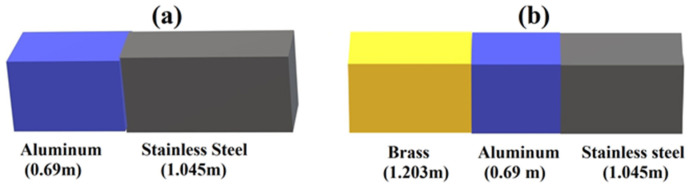
Sketch, not to scale, of two (**a**) and three (**b**) bars in contact. The acoustic spectrum of each bar is characterized and then compared with the one we obtained, putting the bars in contact.

**Figure 6:**
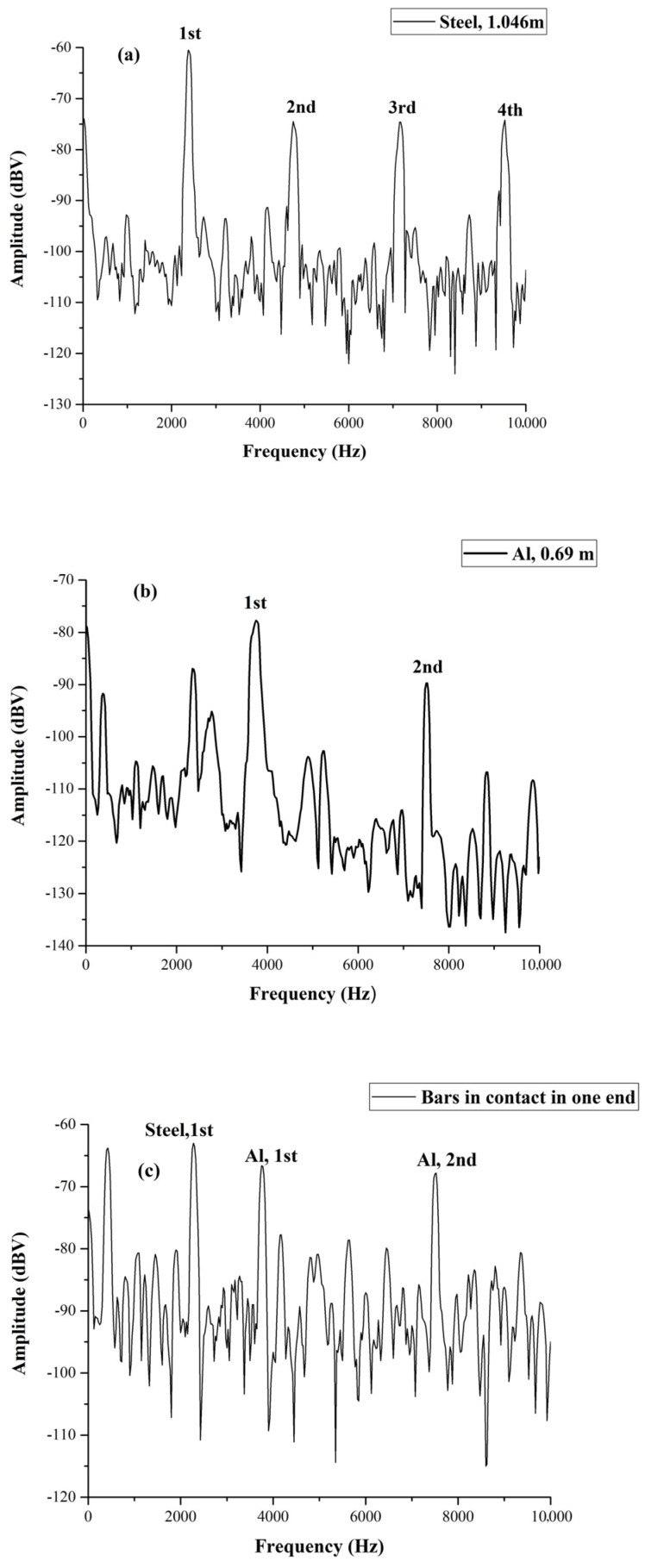
Spectrum of single stainless steel (**a**) and anticorodal aluminum (**b**). In (**c**), we show the spectrum obtained with the two bars in contact at one end. We can see clearly both the 1st harmonic of steel and aluminum. The second harmonic of the aluminum bar is also visible.

Another set of experiments was performed in order to test the capability of our measuring protocol to detect defects by wave reflections and resonances. This second type of experiment consisted of aligning metallic bars and putting them in contact through their ends, as shown in Figure 5a for two bars and in Figure 5b for three bars; the idea is that the discontinuities generated at the contact between the bars mimic a defect. Let us discuss first the experiment performed with two bars for which we used two bars with 4 cm side square sections, one of stainless steel and another of anticorodal aluminum. The stainless steel bar was 1.046 m long while the anticorodal aluminum bar was 0.69 m long, and their acoustic spectra are shown, respectively, in Figure 6a,b. We see that the fundamental frequency of excitation of the stainless steel bar was 2400 Hz, which corresponds to a sound velocity of 5030 m/s, while the anticorodal aluminum bar’s fundamental frequency was 3740 Hz, corresponding to a speed of sound of 5161 m/s. Again, we note the slight differences in these values with respect to those reported in the table. This indicates that it is fundamental for comparative measurements, like the one we present, to characterize all the bars and determine their specific sound velocity one by one.

**Figure 7:**
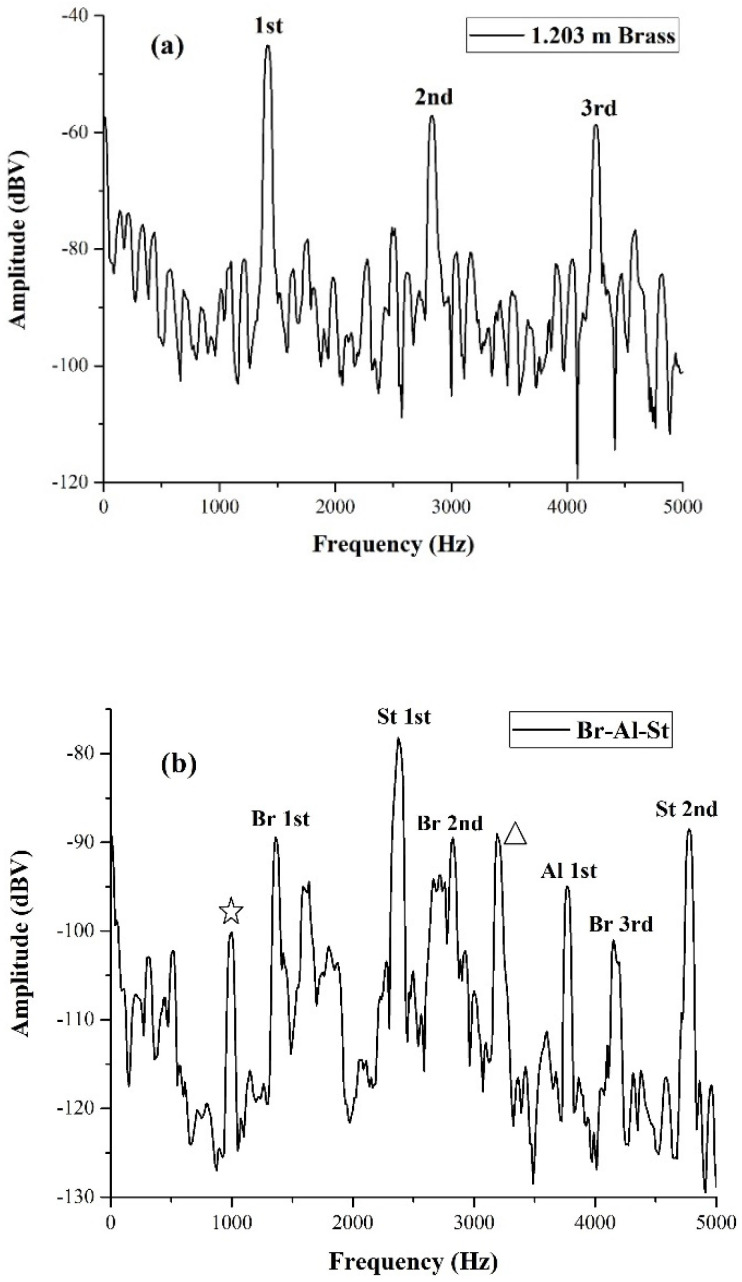
(**a**) Excitations of the isolated brass section of the three bars system of Figure 5b; (**b**) Merging the spectrum in (**a**) with those of the steel and the aluminum, we obtain the spectrum of the entire system corresponding to the three bars in contact. In this case, a slight percussive excitation was applied only to the steel side, and the piezo sensor was positioned on the brass bar. We can see that the spectrum contains the three fundamental frequencies of each of the three bars shown in (**a**) and Figure 6a,b, along with their harmonics.

For the experiment epitomized in Figure 6, the piezo sensor was secured to the aluminum bar, but the light percussive excitation was applied to the steel bar. We ensured that the two bars were in contact by testing the electrical continuity between them. Thus, even if the percussive excitation was applied on the stainless steel bar, oscillations originated, even on the side of the aluminum. In order to make sure that our measurement protocol could allow multiple localizations of defects or discontinuities, we aligned the three bars, as shown in Figure 5b. Here, the aluminum and steel bars were the same ones used in Figure 6. The spectrum of the brass bar is shown in Figure 7a.

The spectrum of the three bars in contact shows three spectral components of brass, the first harmonic of aluminum, and two harmonics of steel. Moreover, since the sound velocities in stainless steel and aluminum are very close (see Table 1), it is likely that the starred spectral component 25 dbV above the noise at 1000 Hz is due to a resonance developing all over the aluminum and steel bars (total length 2.248 m). This resonance might be more evident than in Figure 7 because the reflections at the contact between aluminum and brass (having substantially different propagation velocities) bars can enhance reflections. Note that a harmonic of this resonant frequency (the third, identified by a triangle) is also evident slightly above 3000 Hz; it is possible that the second harmonic does not develop due to the boundary conditions between brass and aluminum.

## 4. Discussion and Applications

There are several aspects of safety and stability in the engineering of structures where the techniques we discussed, namely, detecting acoustic excitations by piezo sensors, could be useful. These aspects range from detecting improper installations in constructions to testing the status of conservation of strands for bridges and cable cars or testing the stability, and possible defects, in train tracks. All these would naturally require protocols that should be set up for each specific case. The “resonant detection” protocol that we have herein discussed could be useful when the length of metals to be tested is “moderate”. Alternative experimental approaches could be sought, based on our detection technique, to check the status of metal bars or strands when their length becomes large (of the order of tens of kilometers or more).

**Figure 8:**
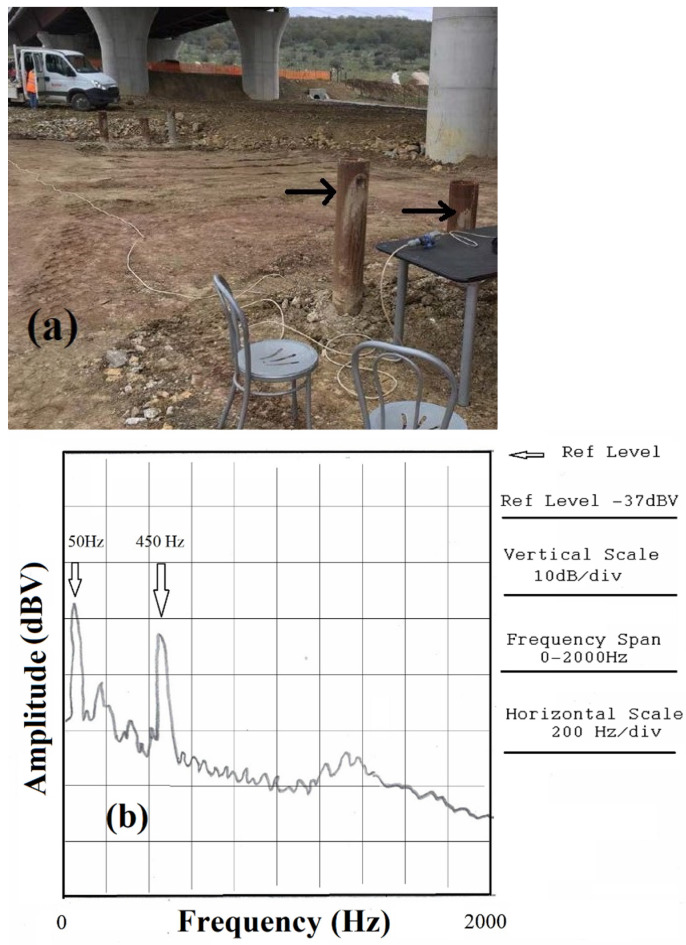
(**a**) Testing the length of steel piles (indicated by arrows) on a road construction site. The piles that we see buried in the ground (and filled with concrete) are supposed to be 12 m long; (**b**) acoustic spectrum of a pile where we clearly see the second harmonic at 450 Hz. From this value, we calculate the length of the pile to be 11.87 m, which is within 1% of the expected 12 m value. The 50 Hz component visible in the spectrum is due to the electrical power line. (**c**) Typical fasteners for iron bars used in foundation cages. Details of the vertical scale are indicated on the right side.

We first show an example of testing structures by the “resonance” approach that we have performed on a road construction site, namely, the Azienda Nazionale Autonoma delle Strade Statali (ANAS) worksite on the highway SS675 Orte-Civitavecchia, located in Vetralla (Italy). For this site, steel foundation piles, like those indicated by the arrows in Figure 8a, were employed for the stability of structures. One of the problems related to this kind of pile when investigating a posteriori the properties of constructions is to make sure that its length is effectively the one estimated for the stability of the served structures. As we see in the figure, the piles are buried in the ground (and then filled with concrete), and it would be difficult to check the physical properties of the piles, such as length, once they are set into operation.

The speed of sound propagation velocity of the steel piles used on the roadsite we are considering was measured in one test pile to be 0.87 m long with a diameter of 0.2 m and a steel thickness of 0.015 m. The value resulting from the acoustic excitation spectrum in the laboratory was 5340 m/s, which is within the range of velocities (see Table 1) expected for steel. It is worth noting that since the pipe we used for the test was empty, we had to fill it with sound-absorbing material in order to dampen the acoustic modes that could develop inside the cavity. This does not represent a problem for the piles filled with concrete, however, due to the fact that at one end, the pile is not free but buried in the ground, differences from a straight, free end, vibrating model, like the one illustrated in Figure 1a may exist. Indeed, the acoustic spectrum we obtained from one pile is shown in Figure 8b, and from it, we can clearly see only the second harmonic (roughly 20 dB above the noise level) of the oscillation pattern at 450 Hz. From this frequency, we calculate a sound velocity of 5340 m/s and a length of the pile of 11.87 m, which is within 1% of the expected value of 12 m. Thus, in this case, our approach provides an excellent estimate of the length of the buried pipe. In this case, the piezo sensor assembly was secured by tape to the pipe to the exposed side, and a slight percussive excitation was applied at the top of the pipe.

**Figure 9:**
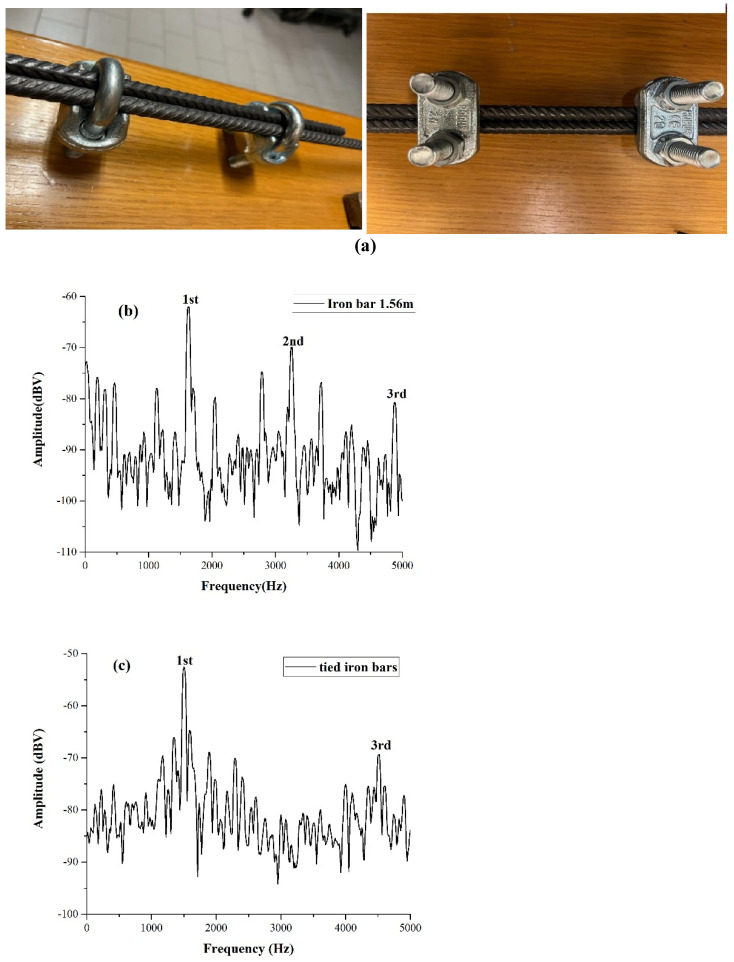
(**a**) Two iron bars tightened together to form a 1.56 m long bar; (**b**) the spectrum of a single iron bar 1.56 m long; (**c**) the spectrum of the tightened bars showing the first harmonic at the same frequency as the single bar in (**b**).

The other example we discuss concerns tests that we performed on an ANAS road construction site for highway SS79 located in Colli sul Velino (Italy). In this site, “iron cages” assembled with cylindrical iron bars 2 cm in diameter are placed inside foundation potholes and then buried in concrete. ANAS provided some iron bars of the same “batch” of those used on the site, and we determined, in the laboratory, the speed of sound to be 1.5 m long in samples, which had a value of 5000 m/s. Two iron cages, each 15 m long and 1 m wide, were put on top of each other and, their ends were tied together by robust iron alloy fasteners, as shown in Figure 9a. Once the cages had been positioned in the foundation pothole, it was filled with concrete. The percussive excitation at the “exposed” ends of the iron bars allowed us to calculate a first harmonic frequency of 85 Hz, not far from the value 83 Hz expected from a 30 m long bar considering a speed of sound of 5000 m/s.

In the laboratory, we tested indeed that when two bars are firmly secured to each other, the fundamental frequency corresponds to oscillations taking place over the total length of the two bars. An example of this kind of test is shown in Figure 9. In (a), we can see two views of the system (tightening tools) used to secure the bars to each other in construction engineering. The bars are passed through the tools and then firmly tightened to each other by the screws. In Figure 9b, we show a spectrum of a single 1.56 m iron bar, the first harmonic at 1620 Hz, and the successive harmonics at 3260 Hz and 4880 Hz. Then, tightening two bars together, we made a bar with a total length of 1.56 m, and the spectrum of it is shown in Figure 9c. We see that the first harmonic of this system appears at 1510 Hz, and the third is at 4500 Hz. These two values are within 7% of the frequency of the single bar of the same length; however, it is likely that for longer lengths, the difference in frequency between a single bar and two tie bars of the same length can be substantially lower (as was the case at the ANAS Colli sul Velino site). Note that the two tied bars in Figure 9c had lengths of 1.18 m and 0.82 m, respectively, and the resonant frequencies corresponding to these values could be expected at 2745 Hz and 3951 Hz, respectively, but there is no trace of these spectral lines in Figure 9c. Thus, the spectral lines that we observe correspond to oscillations taking place over the total length of the two tied bars.

Nevertheless, we also observed that when two iron bars are tied to each other by wires twisted around them or strong tape, it is rather difficult, almost impossible in reality, to observe frequencies corresponding to oscillations that take place over the whole length of the two tied bars. Thus, our measurement protocol cannot provide reliable information on the length of the buried iron bars of the cage when the bars are not firmly tightened, a condition always recommended by structural engineers. Moreover, in Figure 8b, we can clearly see the power supply spectral component at 50 Hz. On the construction sites, we realized that, considering the low power of the percussive excitations and signal amplitude (roughly −70 dBV in Figure 8b), work will be required to set noise reduction at the input of the FFT analyzer. In this respect, we must say that, in addition to more “classical” techniques [23], a new generation of approaches to the problem of noise reduction, based on machine learning and artificial intelligence, are being pursued [24], and we are confident that specific noise filtering could be worked out for developments of our type of measurements.

## 5. Conclusions

We have presented results showing that it is possible to determine characteristic physical features of bars, pipes, and strands using a technique based on the excitation of autonomous resonances in metals. We have shown that wave/pulse propagation in the bars is very analogous to similarly observed phenomena in wave dynamics, in particular for what concerns transmission and reflections at given boundaries of defect scattering.

From the applied point of view, we have seen that the phenomenon offers the possibility to characterize metallic bars when they are not accessible over their whole length. We have shown that the low-frequency autonomous oscillations generated in these elements by light percussive excitations and detected by sensitive piezo sensors can enable gathering information on the properties of the considered elements, such as length, defects, and status of conservation. Our method relies on the possibility of testing “reference” samples, on which the sound propagation velocity can be safely determined a priori. The “reference” samples must be made of the same materials as the ones under investigation. The results that we obtained from measurements on road site constructions are very encouraging, and we are confident that a development/engineering of our methods could provide useful diagnostic tools. The spectra of the signals that we obtain exhibit components whose origins are identifiable and whose amplitudes are tens of dBVs above noise levels. The frequency reading that we report corresponds to the values we read on the LabView 2019 acquisition software (available from the software repository of University of Roma Tor Vergata) however, the error related to those can be estimated from the linewidths of the spectral lines.

We have tested our experimental technique in the laboratory, even on iron strands, which represent important elements in construction. These elements attract much attention, and testing their state of health by piezo sensors has also been considered and recently collected in a rather comprehensive review, which we have referenced. We have not tested the specific methodology we propose on post-tensioned tendons at work on construction sites but, from the data we have presented, and considering the present problems in the literature, we believe one could attempt, using our approach, to investigate the state of post-tensioned tendons on construction sites where the tendons are part of constructed structures.

Our technique relies on the application of very light percussive excitations (of the order of 0.1 newtons, corresponding to a few mJ of energy), and one must make sure, when measurements are performed, that there are no other phenomena/excitations that can perturb the response of the sensitive piezo sensors; further development of our idea should consider the possibility to work in “noisy” environments. We are confident that “well established techniques” and recent developments will help a lot in this respect.

We have provided examples that our approach can be useful for determining the length of iron/steel buried in concrete foundations, which is a relevant problem within the field of construction control and safety. There exist other fields within the framework of structures security; our detection protocol is based on sensitive piezo sensors sticking on metals, and the generation of “autonomous” acoustic excitations could be employed. When the status of very long metallic “bars” or iron strands should be tested, the approach herein presented, based on resonances and the spectrum of induced oscillations, could be taken as a basis to provide other detection principles and new measurement protocols.

## Figures and Tables

**Table 1:** Measured characteristics of the sound velocity of materials determined from the acoustic spectrum of cylindrical bars (reference values have been “averaged” from Refs. [21,22] and other sources).

Bar/Strand Material	Length (Meters)	Sect. Diam. (cm)	Measured Velocity (m/s)	Expected Velocity (m/s)
Aluminum	1.89	2	4913	5100
Brass	1.89	2	3600	4300
Copper	1.89	1.2	3830	3570
Iron	1.565	2	5130	5000
Steel	1.89	2	4900	5000–5900
Iron Strand	1.89	2	5560	-
1m Iron Strand	1	2	5446	-

## Data Availability

The data presented in this study are available upon request from the corresponding author.
